# Isolated emergency medical incidents in the practice of Polish firefighters in 2020–2023: analysis of intervention causes

**DOI:** 10.3389/fpubh.2025.1721695

**Published:** 2025-12-16

**Authors:** Łukasz Dudziński, Tomasz Kubiak, Robert Gałązkowski, Julia Grochowska, Łukasz Czyżewski, Attila Pandur

**Affiliations:** 1 Department of Medical Rescue, Medical University of Warsaw, Warsaw, Poland; 2 Department of Health Sciences, Poznan Medical Academy of Applied Sciences Mieszko I, Poznan, Poland; 3 Department of Agricultural Science, John Paul II University in Biala Podlaska, Biala Podlaska, Poland; 4 Department of Geriatric Nursing, Medical University of Warsaw, Warsaw, Poland; 5 Department of Oxyology and Emergency Care, Institute of Emergency Care, Pedagogy of Health and Nursing Sciences, Faculty of Health Sciences, University of Pecs, Pécs, Hungary

**Keywords:** firefighting and rescue operations, EMS support, IEMI, qualified first aid, NFRS

## Abstract

**Objective:**

To quantify and characterize Isolated Emergency Medical Incidents (IEMIs) managed by National Firefighting and Rescue System (NFRS) units in Poland in 2020–2023 and to identify temporal and regional patterns.

**Materials and methods:**

Retrospective nationwide analysis of anonymised State Fire Service decision-support records. Inclusion required NFRS arrival before EMS, documented direct actions, duration over 1 min, and dates 01.01.2020–31.12.2023. Descriptive statistics were compiled at the voivodeship level. Group differences were tested with Kruskal–Wallis and Dunn–Bonferroni procedures. Associations were examined with Spearman correlation. Visualizations included heat maps, box plots, and a choropleth. Seasonality was assessed by calendar quarter.

**Results:**

Approximately 2.25 million interventions were undertaken; medical rescue comprised about 35%. Regional heterogeneity was significant (χ^2^ = 46.72; *p* < 0.001). Mean IEMI duration differed by year (χ^2^ = 28.35; *p* < 0.001), peaking in 2021 and declining in 2022–2023 (medians 13–12 min). Pairwise contrasts showed 2020 > 2022 (*p* = 0.015), 2020 > 2023 (*p* = 0.001), 2021 > 2022 (*p* = 0.001), and 2021 > 2023 (*p* < 0.001). Minimum time did not correlate with the mean (*ρ* = 0.05; *p* = 0.7); maximum time did (*ρ* = 0.55; *p* < 0.001). Incident counts correlated with population (ρ = 0.518) and urbanization (ρ = 0.446), modestly with number of EMS teams (*ρ* = 0.396), and weakly with area (ρ = 0.277). Q4 increases were consistent for cardiac arrest, unconsciousness, and dyspnoea; hemorrhage, choking, psychiatric disorders, and other showed no seasonality. Annual totals did not differ (H(3) = 1.83; *p* = 0.608).

**Conclusion:**

IEMIs form a stable share of NFRS activity, dominated by cardiac arrest, syncope, trauma, and neurological disorders, with a Q4 peak. Seasonal readiness in October–December, targeted QFA training on CPR/AED, resource allocation by per capita and urbanization metrics, early activation thresholds, standardized winter workflows, and quarterly quality monitoring may improve timeliness and reduce demand.

## Introduction

The State Emergency Medical Services (EMS) were established to fulfill the state mandate to protect the life and health of citizens. Units within this system include Hospital Emergency Departments (EDs) and EMS units, including the Helicopter Emergency Medical Service (HEMS). Numerous entities cooperate with EMS, including units of the National Firefighting and Rescue System (NFRS), namely the professional Fire Service (FS) supported by the Volunteer Fire Brigade (VFB) (1–3).

The mission of firefighters is to protect and save life and health, safeguard the environment, and anticipate, identify, and mitigate hazards. Because saving the public is the priority, firefighters implement medical rescue procedures. In Poland, this component of rescue is based on procedures and training in Qualified First Aid (QFA). Through QFA, firefighters provide the main operational support to medical entities. Under established dispatch rules, firefighters are deployed to two main categories of events: fires (F) and non-fire incidents referred to as local threats (LT). In both categories, firefighters are prepared to perform QFA procedures. The same procedural standards and equipment are used across all fire service units (4, 5).

An Isolated Emergency Medical Incident (IEMI) handled by NFRS units is an intervention intended for EMS when ambulances are unavailable or have a longer response time. In such situations, rapid firefighter intervention is driven by the time-critical nature of the patient’s life-threatening condition. Under dispatch protocols, an event may also meet the features of an IEMI if firefighters directly observe a health emergency, for example while traveling in a fire engine from another incident or from training. In IEMIs, the ambulance response time is longer than the firefighters’ response time (6, 7).

Multiple factors can locally prolong EMS arrival times, including demographic and environmental features, population density, mass-casualty situations, the simultaneous deployment of all medical resources to other events, or less favorable positioning of medical units compared with the location of fire units relative to the incident site (8).

Specialized Rescue Units (SRUs) operate within Polish fire service response structures and cover various specializations, such as water and diving, medical rescue, search and rescue, technical, and high-angle operations. All units, at both basic and specialized levels, provide medical assistance at the QFA level. Specialist operations in the hazard zone constitute IEMIs because on-scene medical services initially lack access to the casualty. Firefighters can intervene within the hazard zone and, during evacuation from that zone, perform medical procedures such as airway management and hemorrhage control. These critical actions enable medical teams to continue care outside the hazard area. EMS entities generally lack personal protective equipment at the level used by firefighters and lack procedures for work inside such zones, which may contain casualties. Firefighter actions undertaken within the hazard zone can therefore improve patient prognosis (9, 10).

As a cooperating service with EMS, firefighters handle IEMIs directly at the patient’s side on a stand-alone basis, provide auxiliary tasks in cooperation with medical services, or perform non-medical support for EMS entities, including technical and specialist operations, patient transfer, scene lighting, and access provision. All of these supporting activities influence the effectiveness of medical care and patient outcomes (11, 12).

This study provides, to our knowledge, the first nationwide description of IEMIs in Poland with concurrent temporal and spatial analysis. By quantifying seasonality and regional heterogeneity across clinically salient categories, we identify modifiable operational levers for NFRS–EMS coordination and training. According to available scientific reports and official website sources, in many European countries, firefighters support medical teams or have medical equipment. The medical procedures of Polish firefighters and the results presented in this study confirm this trend (13–15).

## Objective

To conduct a retrospective review and presentation of the reasons for dispatching NFRS units to interventions classified as IEMIs across Poland in 2020–2023.

## Materials and methods

### Design

This is an observational, retrospective descriptive study. Observations of quantitative data concerned interventions of NFRS units based on data from the Decision Support System (DSS) from the years 2020–2023. The analysis was based on the data for the whole country (Poland). The database includes: total number of interventions, causes divided into categories (Unconsciousness, Fainting, Cardiac Arrest, Dyspnoea, Acute Coronary Syndrome, Hemorrhage, Choking, Bodily Injuries, Psychiatric Disorders, Neurological Disorders, Other), time of intervention, division into Polish regions (voivodeships) in relation to financial conditions and population density, quarters of the year.

### Project

Data for analysis were provided by the Operational Planning Bureau (BPO) with the approval of the Main Headquarters of the SFS. Approval was granted in May 2023. Observations cover the years 2020–2023 across Poland. We used data from the Decision Support System of the State Fire Service (DSS-SFS), which archives all firefighter interventions. The quantitative analysis is fully anonymized for all individuals involved in the incidents (addresses, event dates, service personnel, casualty data, vehicle identification numbers). Therefore, no application was submitted to an ethics committee.

### Statistical analysis

Statistical analyses were performed using Statistica version 13 (TIBCO Software Inc., Palo Alto, CA, United States). The significance level was set at 0.05. Normality was tested with the Shapiro–Wilk test; because a substantial proportion of quantitative variables deviated from normality, nonparametric tests were applied. Descriptive statistics were calculated for annual sums of incidents across voivodeships for 2020–2023, including N, minimum, maximum, sum, mean (M), standard deviation (SD), median, and interquartile range (IQR). Group differences were assessed with the Kruskal–Wallis test and Dunn–Bonferroni *post hoc* correction. A heat map was generated to visualize differences in the number of incidents across specific categories in individual voivodeships over the study period. The average incident duration between years was compared using the Kruskal–Wallis test, with results shown as box plots. The influence of minimum and maximum time on the mean was examined using Spearman rank correlation. Correlations between the number of incidents and voivodeship area, population size, number of emergency medical teams, and degree of urbanization were also assessed with Spearman’s method, with results presented as a heat map. A choropleth map (cartogram) illustrated the incident index per 1,000 inhabitants in each voivodeship. Finally, seasonality of specific incident types was analyzed by comparing quarterly frequencies using the Kruskal–Wallis test with Dunn–Bonferroni correction. Year-to-year comparisons of the number of interventions were performed based on annual sums for 16 voivodeships across the four study years.

### Inclusion criteria

Incidents were included if fire service units intervened with the casualty before EMS entities arrived and if direct actions at the casualty were recorded in DSS-SFS. The observation window was 01.01.2020 (00:00) to 31.12.2023 (23:59). Only incidents lasting more than 1 min were analyzed. Under operational timekeeping and dispatch rules, an IEMI ends when EMS units arrive and assume command and responsibility for the casualty. Events with IEMI characteristics in which firefighters arrived first but medical services reached the scene within one minute, despite DSS-SFS classifying them as IEMIs, were excluded because this interval does not reflect meaningful, independent firefighter actions. After EMS arrival, firefighters typically remain on scene in a supporting role.

### Ethical considerations

The cases described are fully anonymous, retrospective, the analysis complies with the principles of the Declaration of Helsinki and did not require any approval of a bioethics committee.

## Results

Across the study period, NFRS entities undertook a total of approximately 2.25 million interventions: 2020, 583.2 thousand; 2021, 579.7 thousand; 2022, 608.8 thousand; 2023, 486.1 thousand. Medical rescue operations accounted for about 35% of all interventions. Within this subset were IEMIs and joint operations with EMS for acute life-threatening emergencies (Table 1).

**Table 1:** General characteristics of IEMI causes in 2020–2023.

Variables	N	MIN	MAX	SUM	M	SD	MDN	IQR	p
Unconsciousness	64	1	93	1292	20.19	18.17	15	17.25	<0.001
Fainting	64	2	238	3775	58.98	49.88	41	63.5	
Cardiac arrest	64	10	814	6452	100.81	125.8	66	80	
Dyspnoea	64	0	80	1211	18.92	21	9.5	24.25	
Acute coronary syndrome	64	0	42	533	8.33	9.69	4.5	8	
Hemorrhage	64	0	66	890	13.91	13.48	9	16	
Choking	64	0	15	154	2.41	3.31	1	3.25	
Bodily injuries	64	1	211	2680	41.88	40.05	30	45.25	
Psychiatric disorders	64	0	24	191	2.98	4.21	2	2	
Neurological disorders	64	1	70	1306	20.41	16.73	16	22	
Other	64	0	223	2353	36.77	44.72	22	40.5	

min - minimum, max - maximum, M - mean, SD - standard deviation, MDN - median, IQR - interquartile range.

**Figure 1:**
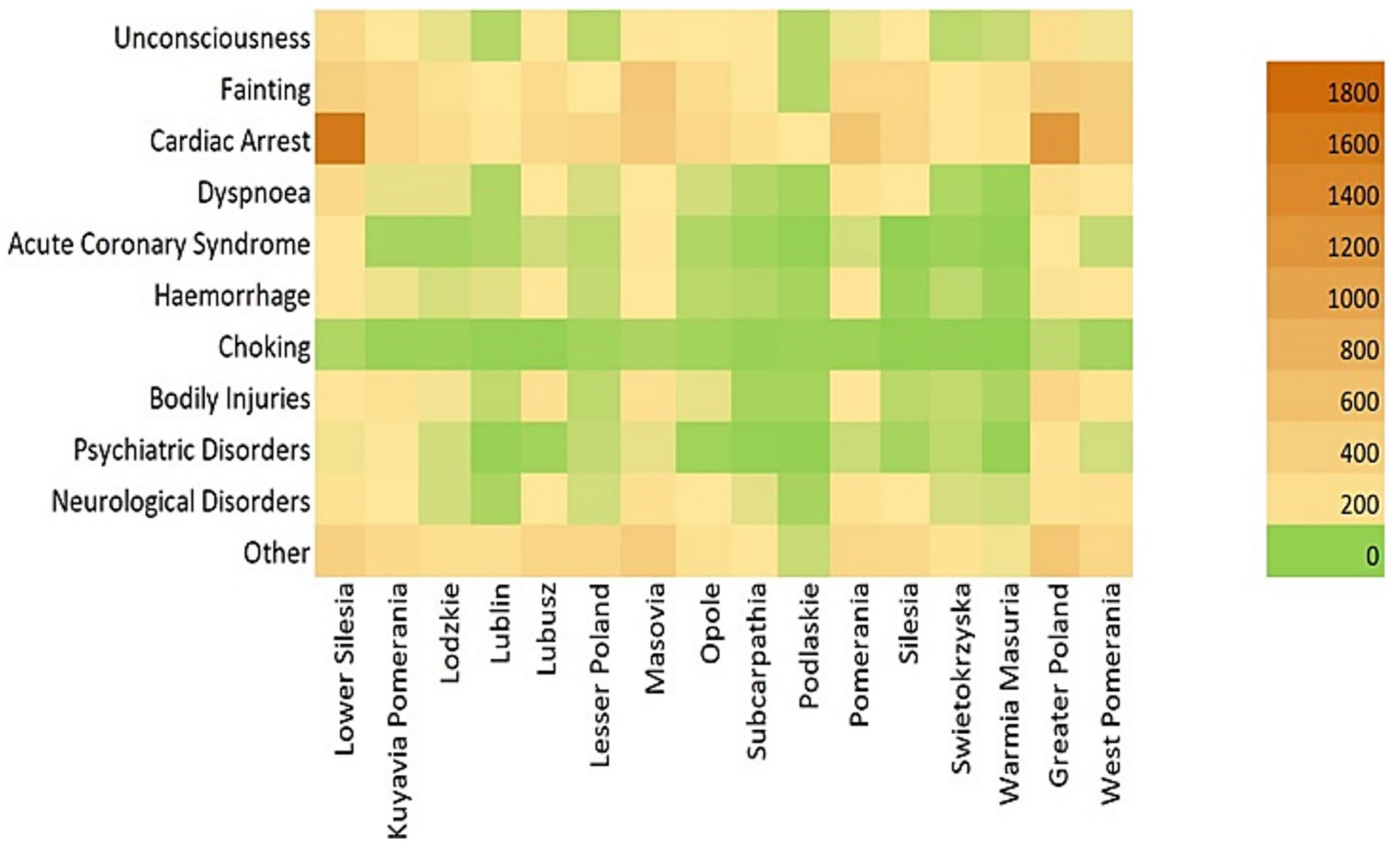
Correlation between interventions and Polish regions.

Our observations cover 2020–2023. According to Main Headquarters of the SFS data, in 2024 there were 7,484 IEMIs, indicating an upward trend (Figure 1).

**Figure 2:**
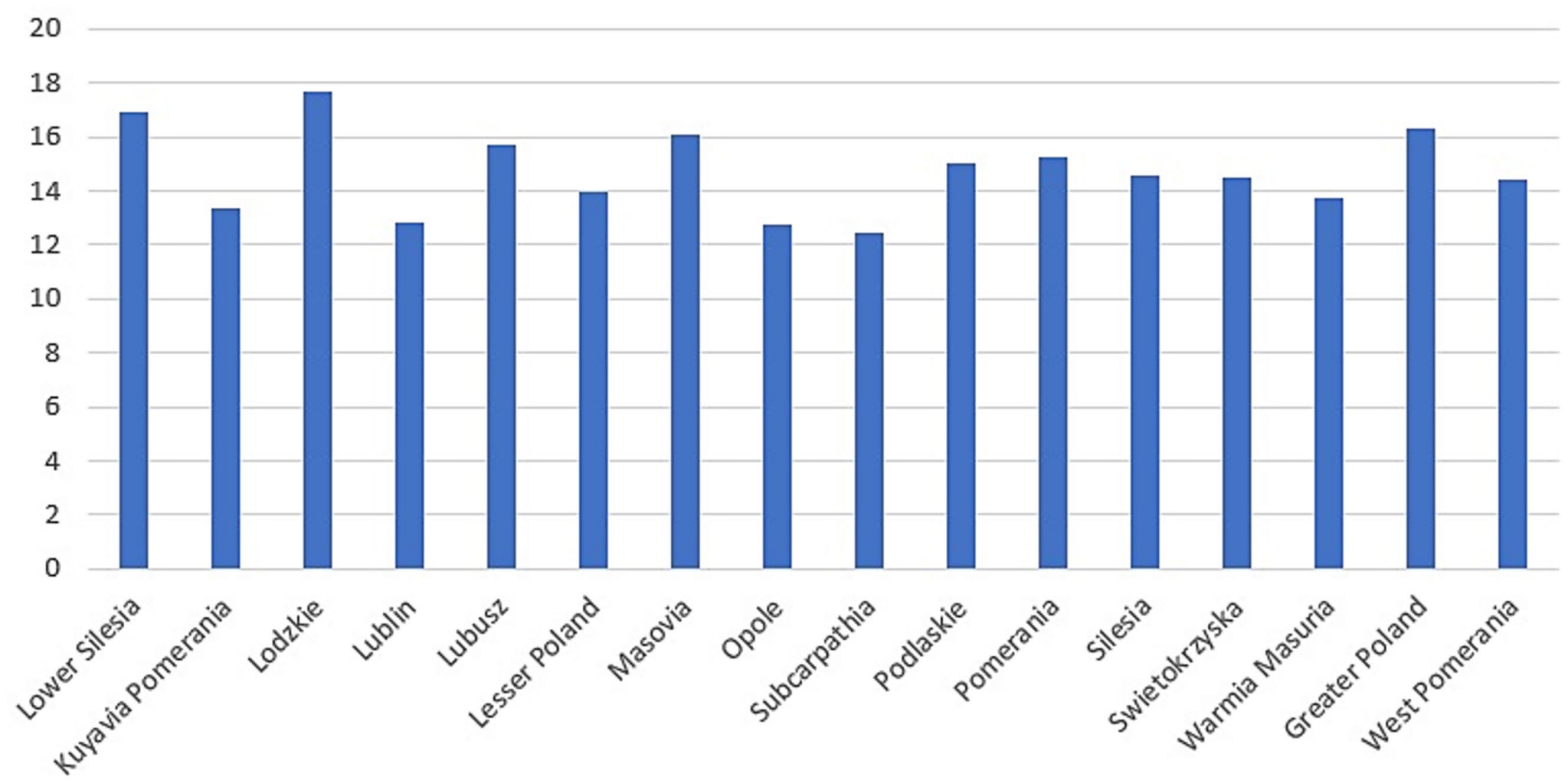
Mean time by voivodeship (minutes).

Regional differences are evident across voivodeships. Lower Silesia shows a particularly high number of interventions, most frequently related to cardiac arrest; Greater Poland, Pomerania, and Masovia also report elevated figures in this category. In contrast, Podlaskie and Warmia-Masuria record fewer interventions across most categories, reflected by the predominance of green shades on the heat map. To assess regional variation statistically, the Kruskal-Wallis test (χ^2^ = 46.72; *p* < 0.001) confirmed significant differences in the number of interventions across voivodeships. *Post hoc* analysis using the Dunn-Bonferroni test identified significant pairwise differences for the following comparisons: Lower Silesia vs. Lublin, Lower Silesia vs. Podlaskie, Lublin vs. Greater Poland, and Podlaskie vs. Greater Poland (Figure 2).

**Figure 3:**
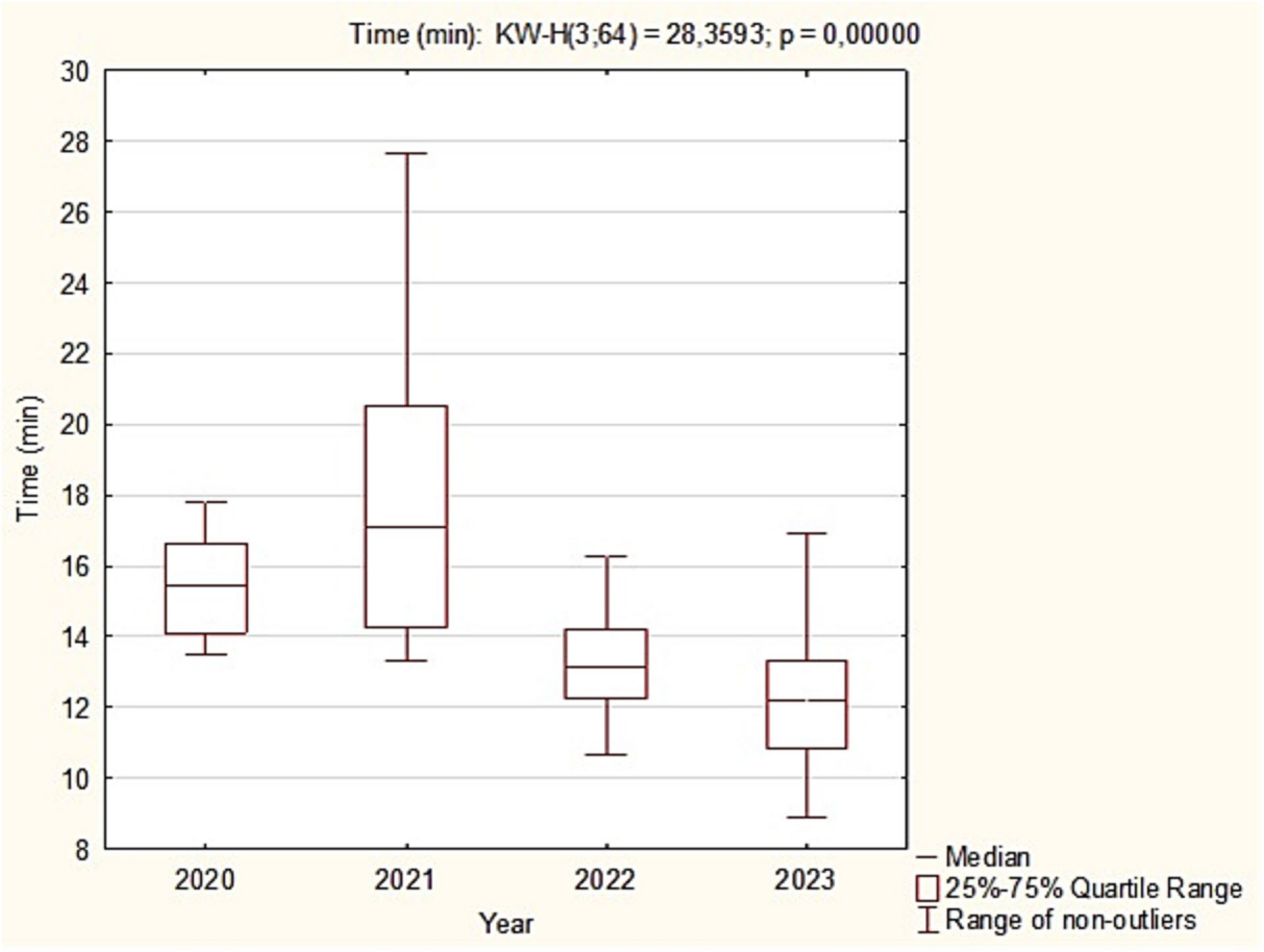
Box plots of mean incident duration (min) by study year, 2020–2023.

The mean time in minutes differed significantly between years (Kruskal-Wallis: χ^2^ = 28.35; *p* < 0.001). The longest times occurred in 2021, as seen in the higher median and a wide range extending beyond 25 min. Times then declined; in 2022 and 2023 the medians were about 13–12 min, and the maxima were clearly lower than in 2021. *Post hoc* Dunn-Bonferroni tests showed: 2020 vs. 2022, significant (*p* = 0.015), with 2020 higher; 2020 vs. 2023, highly significant (*p* = 0.001), with 2020 higher; 2021 vs. 2022, highly significant (*p* = 0.001), with 2021 higher; 2021 vs. 2023, highly significant (*p* < 0.001), with 2021 higher. No significant difference was observed between 2020 and 2021, and the 2022 vs. 2023 difference was not significant (Figure 3).

We also examined associations between minimum and mean times and between maximum and mean times. Spearman correlation showed no association between the minimum and mean intervention times (r = 0.05; *p* = 0.7), indicating that the shortest actions do not affect the average duration. In contrast, a moderate positive correlation was found between the maximum and mean times (r = 0.55; *p* < 0.001), suggesting that very prolonged single interventions raise the overall mean.

**Figure 4:**
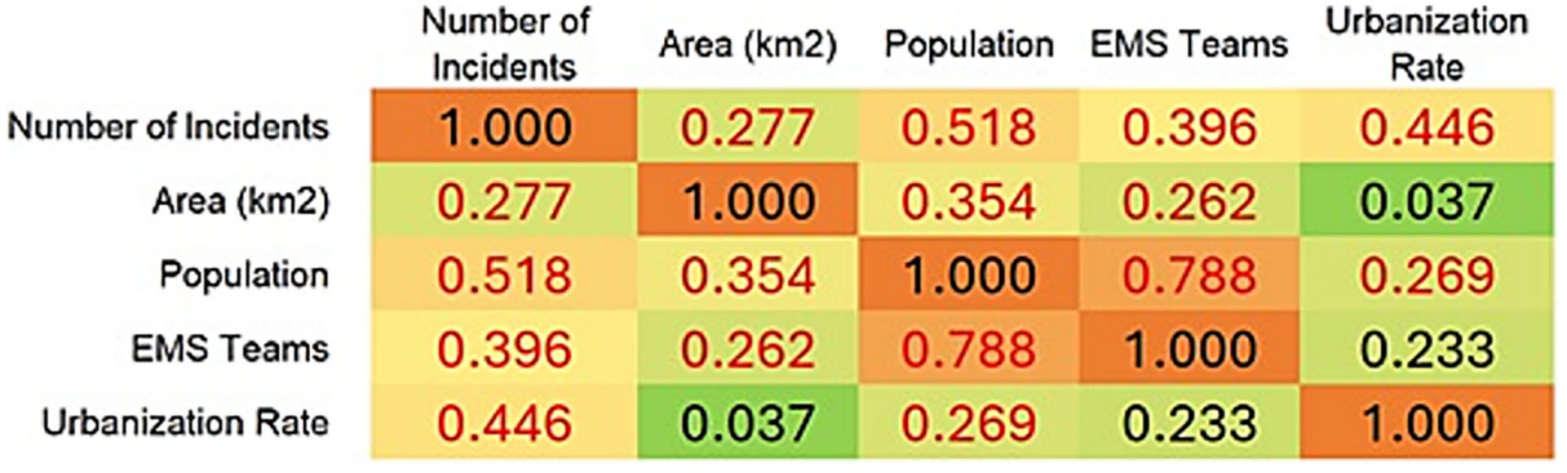
Correlation of incident counts with voivodeship area, population, number of emergency medical teams, and degree of urbanization.

**Figure 5:**
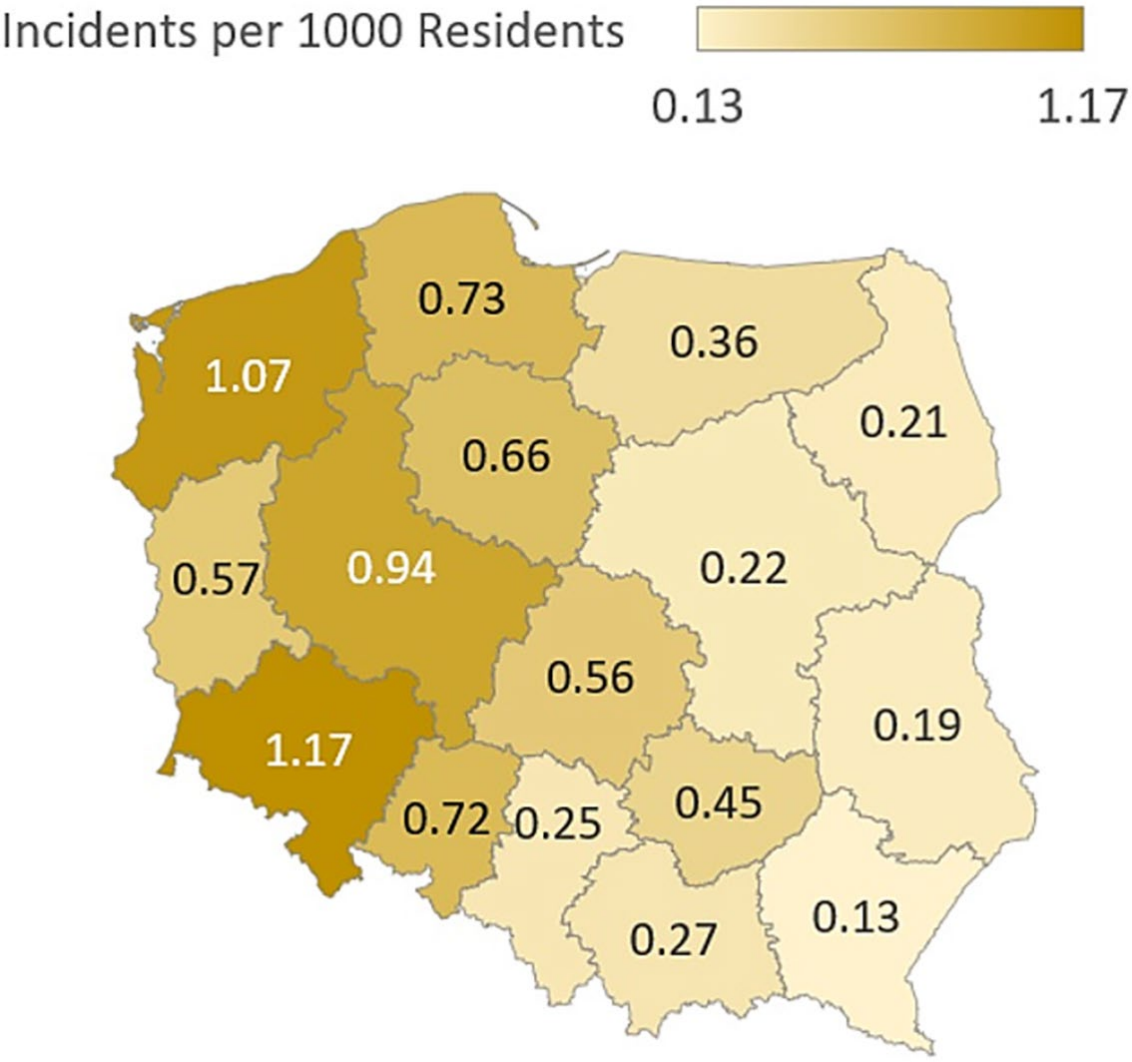
IEMI incidence per 1,000 inhabitants.

The next summaries (Figures 4, 5) were prepared from our data and reports of Central Statistical Office (16).

Based on the Spearman rank correlation coefficients, several statistically significant relationships (marked in red) emerge for the number of interventions. The strongest positive correlation is with population size (*ρ* = 0.518), indicating that more populous regions experience more incidents requiring emergency response. A further moderate positive correlation appears with the urbanization rate (*ρ* = 0.446), suggesting that more urbanized areas have a higher frequency of interventions. A slightly weaker but still significant positive association is observed with the number of EMS teams (*ρ* = 0.396). This may reflect deployment to areas with greater expected demand or resources, or conversely that more teams contribute to a higher recorded volume of interventions. By contrast, geographical area in square kilometres shows the weakest correlation with the number of interventions (*ρ* = 0.277), underscoring that population density rather than territorial extent is more influential. The very low and non-significant correlation between area and urbanization (ρ = 0.037) supports the independence of urbanization from physical size. The very strong correlation between population and the number of EMS teams (ρ = 0.788) suggests a rational allocation of emergency resources relative to resident counts. In summary, the primary factors associated with intervention numbers are population size and level of urbanization, while geographical area is of marginal importance.

The incident index per 1,000 inhabitants showed heterogeneous burden across regions. Elevated rates in selected voivodeships may reflect a higher concentration of high-risk populations or constraints in healthcare infrastructure. This metric is a useful indicator for workforce planning and resource allocation, supporting targeted deployment of NFRS and EMS resources where need is greatest (Table 2).

**Table 2:** Quarterly comparison.

Type of threat	I (M±SD)	II (M±SD)	III (M±SD)	IV (M±SD)	*p*
Unconsciousness	1.61 ± 2.27	1.41 ± 2.14*	1.39 ± 1.74*	2.5 ± 3.73	0.005
Fainting	3.93 ± 4.92*	4.66 ± 5.51*	4.6 ± 4.58	6.99 ± 8.66	<0.001
Cardiac arrest	7.36 ± 8.18*	6.64 ± 7.55*	6.91 ± 9.56*	13.07 ± 31.47	<0.001
Dyspnoea	1.42 ± 2.59*	1.34 ± 3.43*	1.32 ± 1.92	2.46 ± 4.24	0.001
Acute coronary syndrome	1.29 ± 2.43*	1.33 ± 3.44*	1.2 ± 1.87*	2.21 ± 3.4	0.002
Hemorrhage	1.11 ± 1.9	1.1 ± 1.98	1.48 ± 2.37	1.53 ± 2.38	0.064
Choking	0.17 ± 0.46	0.18 ± 0.45	0.21 ± 0.62	0.29 ± 0.77	0.557
Bodily injuries	3.08 ± 3.43*^#^	3.54 ± 3.73*	4.45 ± 3.95	6.27 ± 9.79	<0.001
Psychiatric disorders	0.22 ± 0.74	0.18 ± 0.55	0.22 ± 0.55	0.39 ± 1.75	0.103
Neurological disorders	1.39 ± 2*	1.46 ± 2.02*	1.65 ± 1.93	2,45 ± 3,35	<0.001
Other	2.93 ± 4.14	2.8 ± 3.44	3.02 ± 4.47	5.65 ± 11.3	0.071

* = indicates significance vs Q4; ^#^ = indicates significance vs. Q3.

**Figure 6:**
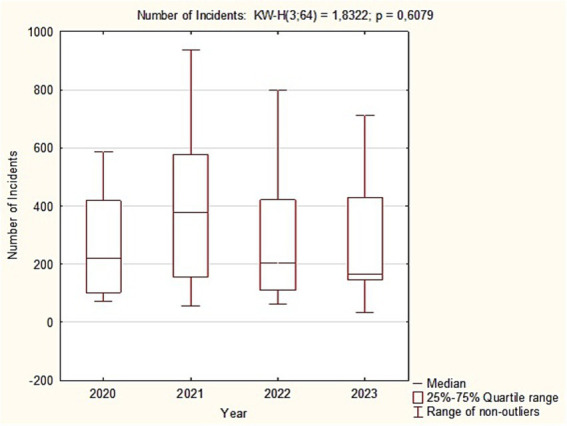
Comparison of annual intervention totals, 2020–2023.

A comparison of incident counts by calendar quarter revealed significant differences for most emergency categories. The highest values most often occurred in Q4. This applied, among others, to Cardiac Arrest (M = 13.07; *p* < 0.001), Unconsciousness (M = 2.50; *p* = 0.005), and Dyspnoea (M = 2.46; *p* = 0.001). Exceptions included Hemorrhage, Choking, Psychiatric Disorders, and Other, for which no seasonal differences were identified. These findings suggest that seasonal factors, such as respiratory infections and winter conditions, substantially contribute to the increased frequency of IEMIs at the end of the year (Figure 6).

Visual inspection of the box plot showed no clear upward or downward trend in the number of medical interventions across the four-year period. The yearly medians were relatively stable, and the interquartile ranges and whiskers overlapped substantially. This interpretation was confirmed by the Kruskal-Wallis test (H(3) = 1.83; *p* = 0.608). The high *p* value provides insufficient evidence to reject the null hypothesis. We therefore conclude that there were no statistically significant differences in the annual number of medical interventions between 2020, 2021, 2022, and 2023.

## Discussion

Firefighters are one of several formations that cooperate with EMS. Beyond firefighters, EMS is supported within defined competencies or areas of operation by the Police, Border Guard, Military, authorized mountain and water rescue services, and the Maritime Search and Rescue service (SAR). Personnel from these services may provide Qualified First Aid (QFA) or the level designated for their formation (17).

Firefighters often arrive first at the scene, a finding reported across multiple regions worldwide. Below, we place published results in the context of Polish conditions, which were the setting of our study. The scope of medical procedures performed by firefighters varies by country and depends on equipment standards, accepted protocols, education and training, and financial and demographic factors. Boland reports that firefighter medical interventions are limited mainly to sudden cardiac arrest (SCA). In Minnesota, USA, analysis of electronic records compared ambulance and firefighter arrival order. Firefighters are dispatched to all medical calls and perform procedures such as intravenous access, oral medication administration, supraglottic airway placement, nebulization, and intramuscular injections (18). During our observation period, Polish firefighters did not carry pharmacotherapy kits.

In Slovakia, official statistics show joint firefighter and medical interventions, especially in road traffic incidents, fires, disasters, and during various exercises. Cooperation also includes professional development, with firefighters undertaking employment-based rotations in prehospital care. Although our study did not analyze training, Polish legislation under the acts on EMS and on Fire Protection mandates joint trainings, drills, and competitions to improve operational quality. Moreover, personnel in fire units often also work in EMS or in hospitals due to their medical education (19, 20).

In France, the fire service employs nurse firefighters. They conduct screenings, assist with diagnostics, obtain nursing histories during duty, and monitor firefighters’ health. While this does not directly address medical incident response, it illustrates how medical qualifications within the fire service can support crisis response (21). Another report describes deploying medically trained fire personnel to provide internal medical coverage due to the health risks inherent in firefighting. The Seine-et-Marne fire department implemented medical support staffed by firefighters with nursing credentials (22). Our dataset did not isolate injuries or risks among firefighters themselves. Nevertheless, Polish procedures allow provision of QFA to a firefighter at the scene before EMS arrival, which by regulation classifies such events as IEMIs. Another publication describes nurse roles within the fire service as an uncommon career pathway (23).

Portugal provides further corroboration of firefighter support for medical services. Firefighters, in addition to firefighting, deliver first aid and urgent prehospital care (24). Analyses from Sweden provide insights into response times. Firefighters frequently arrived before ambulances, often being first on scene with a median of 17 min in 95% of responses, whereas ambulances required almost twice as long. The most common reason for dispatch was out-of-hospital cardiac arrest (OHCA) (25). Two additional Swedish studies align with these findings. Svensson observed the use of firefighters as medical responders while awaiting an ambulance; when cardiopulmonary resuscitation (CPR) was required, firefighters were first on scene (26). The same author reported on the contributions of professional and volunteer firefighters engaged in first aid. Importantly, fire stations are more numerous and geographically dispersed than EMS stations, yielding broader coverage and shorter response times (27). These conditions parallel Poland, where professional firefighters are extensively supported by volunteer units, as noted by Gałązkowski (28). Our data did not specify how many interventions were initiated by the VFB, but in rural settings shorter VFB travel times are likely.

Findings on bystander response to OHCA further support firefighter involvement. In the Netherlands, an automated external defibrillator (AED) is used before ambulance arrival in roughly 60% of OHCAs. This corroborates other studies and our observations on support to EMS by cooperating services, including firefighters and lay responders with AED access (29).

Cardiac arrest remains one of the leading causes of death globally. Firefighters are among the first responders and play a key role in the chain of survival by performing CPR. An Iranian study of firefighter skills found that simulation-based CPR training produced greater improvements in knowledge and quality than workshop-based training. Regular brief simulation refreshers were recommended. These results support our focus on medical procedures performed by firefighters (30).

The critical role of nursing personnel in firefighter medical operations is also highlighted by Brzozowski et al. In prehospital emergencies, medical services work with firefighters supported by nurses. The most frequent pathologies were trauma, carbon monoxide poisoning, chest pain, and cardiac arrest (31). A Polish study from 2025 echoes these results, analyzing firefighter support to EMS in cardiovascular conditions and classifying causes into groups such as fainting, dyspnoea, chest pain, and SCA/CPR (32).

As Sawicki notes, Polish law authorizes firefighters to perform many medical procedures. His study analyzed firefighter knowledge and skills in medical rescue in relation to sociodemographic variables such as education and age, and service variables such as length of service. Evaluative observations of skills are relevant to readiness and effectiveness in real operations. Our results show a rising trend in these events, implying that firefighter medical knowledge should be maintained at the highest feasible baseline level (33).

Wójcik summarizes the legal basis for firefighter QFA competencies. The primary goal of QFA training is to prepare firefighters to aid casualties at the scene until handover to medical teams. The QFA course lasts over 60 h, ends with an examination, and the credential is valid for three years. The operational readiness described there supports the significance of our central study issue, namely IEMIs (34).

Evidence for firefighter involvement in life-threatening emergencies and implementation of medical procedures comes from many parts of the world. The fire service has extensive capabilities in techniques and equipment to mitigate hazards, enabling medical access to casualties and accelerating prehospital care as well as subsequent hospital procedures. Other researchers have reported firefighter roles in medical rescue through:

- Simulated emergency care delivered by firefighters and the use of medical simulation as an educational method (35).
- Analysis of technical and anthropometric data during external chest compressions performed as part of CPR (36).
- Training and cooperation among firefighters, police officers, and paramedics in CPR under suboptimal conditions (37).
- Evaluation of chest compression quality by firefighters (38, 39).

### Limitations

Our study has several limitations. Age and sex of casualties, as well as clinical details, are not available in State Fire Service (SFS) reports, so the analysis is quantitative and spatial and aims to highlight the versatility of firefighter actions beyond their core statutory tasks; moreover, age and sex were not documented in many intervention descriptions. Information on casualty condition, clinical data, the course of interventions, and ICD-10 diagnoses is unavailable (40), as narrative event descriptions are prepared by the Incident Commander, who does not produce medical records; fire service documentation focuses on operational conduct, including principles, tactics, equipment used, prevailing conditions, and constraints. Finally, IEMI data for 2024 were not included; although the total for 2024 is known to the authors at 7,484 cases, those data were excluded because methods of data collection and presentation, variable classification, and cause categorization in the Decision Support System of the State Fire Service (DSS-SFS) changed, preventing statistical comparability with prior years; data for 2024 and subsequent years may therefore serve as material for future secondary analyses.

The epidemiological threat of COVID-19, which coincided with our observation period, was not taken into account and was not the aim of our observations, which may be the subject of further analyses comparing the pre-COVID and post-COVID periods in isolated emergency medical services incidents in the practice of Polish firefighters.

## Conclusion

Analysis of national SFS/NFRS data from 2020 to 2023 confirms that IEMIs constitute a material and stable area of activity, dominated by sudden cardiac arrest (SCA), syncope, trauma, and neurological disorders, with a marked peak in Q4. Implementing seasonal readiness surges in October–December, targeting QFA training toward CPR/AED, respiratory compromise, and trauma, and reallocating resources according to per capita indices and level of urbanization may improve access and timeliness of interventions. Clear thresholds for early SFS activation when predicted EMS arrival times are prolonged, standardization of IEMI with a winter variant, and quarterly quality monitoring (median time, IQR, share of time-intensive events) will support further reductions in operational times. Implementing concise EMS–SFS cooperation algorithms will limit downtime after EMS arrival, and targeted prevention in regions with the highest indices may reduce demand for interventions.

## Data Availability

The datasets presented in this study can be found in online repositories. The names of the repository/repositories and accession number(s) can be made available by the corresponding author.

## References

[1] Act of 8 September 2006 on State Emergency Medical Services. (Dz.U. z 2006 r. nr 191, item. 1410).

[2] Act of 24 August 1991 on the State Fire Service. (Polish Journal of Laws/ Dz. U. 1991 no 88 item. 400).

[3] Regulation of the Minister of Internal Affairs and Administration on 3 July 2017 r. On the detailed organization of the national rescue and fire-fighting system (Dz.U. 2017 item. 1319).

[4] Main Headquarters of the State Fire Service. Principles of Organizing Emergency Medical Services in the National Rescue and Firefighting System. Available online at: www.gov.pl/web/zasady-organizacji-ratownictwa-medycznego-w-ksrg. Warsaw, (2021). (Accessed August 24, 2025).

[5] Main Headquarters of the State Fire Service. Rules for recording events in the National Rescue and Firefighting System. Available online at: www.gov.pl/web/zasady-ewidencji-zdarzen.

[6] Regulation of the Minister of Health of 3 July 2019 on the State Emergency Medical Services Command Support System. (Dz. U. 2019, item. 1310).

[7] Dudziński Ł Glinka M Glinka P. Medical interventions of the fire service during the COVID-19 pandemic in Poland. Crit Care Innov. (2021) 4:23–31. doi: 10.32114/CCI.2021.4.2.23.31

[8] Dudziński Ł Czyżewski Ł Kubiak T. Instrumental airway unblocking in the practice of Polish firefighters - nationwide observation of firefighting and rescue interventions. Emerg Med Serv. (2024) XI:211–9. doi: 10.36740/EmeMS202404102

[9] Podlasiński R Ciekanowski Z Radkevych A. Specialist rescue groups of the state fire service. De Securitate et Defensione. (2022) 8:145–61. doi: 10.34739/dsd.2022.02.11

[10] Regulation of the Minister of Health of 23 December 2019 on medical rescue activities and health services other than medical rescue activities that may be provided by a paramedic. (Dz.U. z 2019 r. item 2478).

[11] Tymiński J. Prehospital cooperation between qualified first aid providers and healthcare professionals. Sci Rep Fire Univ. (2022) 81:89–106. doi: 10.5604/01.3001.0015.8125

[12] Dudziński Ł Glinka M Kubiak T Feltynowski M. Participation of fire protection units in Poland in ensuring the continuous operation of ventilators for home use — a 7-year observation. Med Res J. (2022) 7:215–22. doi: 10.5603/MRJ.a2022.0038

[13] Tamminen JI Hoppu SE Kämäräinen AJJ. Professional firefighter and trained volunteer first-responding units in emergency medical service. Acta Anaesthesiol Scand. (2019) 63:111–6. doi: 10.1111/aas.13224, 30069869

[14] Andréll C Dankiewicz J Todorova L Olanders K Ullén S Friberg H. Firefighters as first-responders in out-of-hospital cardiac arrest – a retrospective study of a time-gain selective dispatch system in the Skåne region, Sweden. Resuscitation. (2022) 179:131–40. doi: 10.1016/j.resuscitation.2022.08.012, 36028144

[15] Brussels Fire Brigade. Available online at: www.be.brussels/en/about-region/structure-and-organisations/administrations-and-institutions-region/brussels-fire-brigade (accessed Nov 9, 2025).

[16] Central Statistical Office: Emergency aid and medical rescue in Poland in: 2022, 2023, 2024. Available online at: www.stat.gov.pl/obszary-tematyczne/zdrowie/zdrowie/pomoc-dorazna-i-ratownictwo-medyczne.html (accessed Sep 5, 2025).

[17] Zwęgliński T Radkowski R. Fire protection units during the COVID epidemic - functioning and tasks in the first months of SARS-CoV virus activity −2. Sci Rep Fire Univ. (2020) 76:93–114. doi: 10.5604/01.3001.0014.5980

[18] Boland LL Satterlee PA Fernstrom KM Hanson KG Desikan P LaCroix BK. Advanced clinical interventions performed by emergency medical responder firefighters prior to ambulance arrival. Prehosp Emerg Care. (2014) 19:96–102. doi: 10.3109/10903127.2014.942477, 25153541

[19] Drotárová J Mesároš M Lošonczi P. Cooperation between the rescue and fire brigade corps and the medical rescue service representing a basis for an effective integrated rescue system in the Slovak Republic. Ann Burns Fire Disasters. (2021) 34:365–71. 35035331 PMC8717901

[20] Bugaj G. Preparedness of the state fire system to actions in case of hazard of a dangerous biological agent in the context of the COVID-19 pandemic. Sci Rep Fire Univ. (2021) 80:89–107. doi: 10.5604/01.3001.0015.6471

[21] Save-Siejak I Chabin V Chazallet F Vande Voorde L. Nurses firefighters in medical fitness at the departemental fire and rescue service. Soins. (2023) 68:57–61. doi: 10.1016/j.soin.2023.08.014, 37778858

[22] Taillade C Ponsoda S. The firefighter nurse, a key player in operational medical support. Soins. (2023) 68:36–40. doi: 10.1016/j.soin.2023.08.009, 37778853

[23] Beach PR Armstrong A. Nurse and firefighter. Nursing. (2023) 53:46–8. doi: 10.1097/01.NURSE.0000923636.81132.2a, 37074282

[24] Viegas C Sousa P Dias M Caetano LA Ribeiro E Carolino E . Bioburden contamination and *Staphylococcus aureus* colonization associated with firefighter's ambulances. Environ Res. (2021) 197:111125. doi: 10.1016/j.envres.2021.111125, 33895113

[25] Nord-Ljungquist H Bohm K Fridlund B Elmqvist C Engström Å. Time that save lives while waiting for ambulance in rural environments. Int Emerg Nurs. (2021) 59:101100. doi: 10.1016/j.ienj.2021.101100, 34781156

[26] Svensson A Elmqvist C Fridlund B Rask M Andersson R Stening K. Using firefighters as medical first responders to shorten response time in rural areas in Sweden. Aust J Rural Health. (2020) 28:6–14. doi: 10.1111/ajr.12599, 32105393

[27] Svensson A Nilsson B Lantz E Bremer A Årestedt K Israelsson J. Response times in rural areas for emergency medical services, fire and rescue services and voluntary first responders during out-of-hospital cardiac arrests. Resusc Plus. (2024) 17:100548. doi: 10.1016/j.resplu.2023.100548, 38292470 PMC10825318

[28] Gałązkowski R Pawlak A Pszczółkowski K. The role of the National Rescue and firefighting system in the National Emergency Medical System functioning in rural areas in Poland. Saf Fire Technol. (2014) 34:15–26. doi: 10.12845/bitp.34.2.2014.1

[29] Stieglis R Verkaik BJ Tan HL Koster RW van Schuppen H van der Werf C. Association between delay to first shock and successful first-shock ventricular fibrillation termination in patients with witnessed out-of-hospital cardiac arrest. Circulation. (2025) 151:235–44. doi: 10.1161/CIRCULATIONAHA.124.069834, 39462804 PMC11872269

[30] Faghihi A Naderi Z Keshtkar MM Nikrouz L Bijani M. A comparison between the effects of simulation of basic CPR training and workshops on firefighters’ knowledge and skills: experimental study. BMC Med Educ. (2024) 24:178. doi: 10.1186/s12909-024-05165-z, 38395870 PMC10893681

[31] Brzozowski S Buliard S Fey G Rigaudiere P Savall A Charles R. Quelles interventions effectuent les Infirmiers Sapeurs-Pompiers? Sante Publique. (2022) 34:709–16. doi: 10.3917/spub.225.0709, 36577669

[32] Dudziński Ł Kubiak T Czyżewski Ł Bihałowicz S. Support for the national medical rescue system by fire protection units in life-threatening situations due to cardiac disorders. Sci Rep Fire Univ. (2025) 93:101–16. doi: 10.5604/01.3001.0054.9901

[33] Sawicki A Chrościcki D. Evaluation of firefighters' knowledge about medical procedures. Crit Care Innov. (2019) 2:27–36. doi: 10.32114/CCI.2019.2.3.27.36

[34] Wójcik G Surowicz D Kuźnicki M. The state of knowledge of qualified first aid among firefighters of the National and Volunteer Fire service. Saf Fire Technol. (2017) 45:102–10. doi: 10.12845/bitp.45.1.2017.8

[35] Abelsson A Lundberg L. Simulation as a means to develop firefighters as emergency care professionals. Int J Occup Saf Ergon. (2019) 25:650–7. doi: 10.1080/10803548.2018.1541122, 30362390

[36] Silva MD Barbieri RA Foresti YF Cursiol JA Viana FA dos Santos EF. Association of training in basic life support with the evolution of cardiopulmonary resuscitation performed by firefighters. Emerg Med Int. (2023) 2023:8150697. doi: 10.1155/2023/8150697, 37188319 PMC10181904

[37] Abelsson A Appelgren J Axelsson C. Low-dose, high-frequency CPR training with feedback for firefighters. Int J Emerg Serv. (2018) 8:64–72. doi: 10.1108/IJES-01-2018-0001

[38] McAlister O Harvey A Currie H McCartney B Adgey J Owens P . Temporal analysis of continuous chest compression rate and depth performed by firefighters during out of hospital cardiac arrest. Resuscitation. (2023) 185:109738. doi: 10.1016/j.resuscitation.2023.109738, 36806652

[39] Dudziński Ł Glinka M Wysocki D Leszczyński P Panczyk M. Quality analysis of chest compression during cardiopulmonary resuscitation performed by firefighters with physical effort. Pol Heart J. (2021) 79:690–2. doi: 10.33963/KP.15992, 33926175

[40] International statistical classification of diseases and related health problems (ICD-10), volume X. Geneva: World Health Organization (2020).

